# Posterior Cortical Cognitive Deficits Are Associated With Structural Brain Alterations in Mild Cognitive Impairment in Parkinson’s Disease

**DOI:** 10.3389/fnagi.2021.668559

**Published:** 2021-05-13

**Authors:** Quentin Devignes, Romain Viard, Nacim Betrouni, Guillaume Carey, Gregory Kuchcinski, Luc Defebvre, Albert F. G. Leentjens, Renaud Lopes, Kathy Dujardin

**Affiliations:** ^1^Lille Neuroscience and Cognition, Lille University, Inserm, Lille University Medical Centre, Lille, France; ^2^US 41—UMS 2014—PLBS, Lille University, CNRS, Inserm, Lille University Medical Centre, Pasteur Institute, Lille, France; ^3^Department of Neuroradiology, Lille University Medical Centre, Lille, France; ^4^Neurology and Movement Disorders Department, Lille University Medical Centre, Lille, France; ^5^Department of Psychiatry, Maastricht University Medical Centre, Maastricht, Netherlands

**Keywords:** volumetry, shape analysis, cortical thickness, image texture analysis, tractometry, cognition

## Abstract

**Context**: Cognitive impairments are common in patients with Parkinson’s disease (PD) and are heterogeneous in their presentation. The “dual syndrome hypothesis” suggests the existence of two distinct subtypes of mild cognitive impairment (MCI) in PD: a frontostriatal subtype with predominant attentional and/or executive deficits and a posterior cortical subtype with predominant visuospatial, memory, and/or language deficits. The latter subtype has been associated with a higher risk of developing dementia.

**Objective**: The objective of this study was to identify structural modifications in cortical and subcortical regions associated with each PD-MCI subtype.

**Methods**: One-hundred and fourteen non-demented PD patients underwent a comprehensive neuropsychological assessment as well as a 3T magnetic resonance imaging scan. Patients were categorized as having no cognitive impairment (*n* = 41) or as having a frontostriatal (*n* = 16), posterior cortical (*n* = 25), or a mixed (*n* = 32) MCI subtype. Cortical regions were analyzed using a surface-based Cortical thickness (CTh) method. In addition, the volumes, shapes, and textures of the caudate nuclei, hippocampi, and thalami were studied. Tractometric analyses were performed on associative and commissural white matter (WM) tracts.

**Results**: There were no between-group differences in volumetric measurements and cortical thickness. Shape analyses revealed more abundant and more extensive deformations fields in the caudate nuclei, hippocampi, and thalami in patients with posterior cortical deficits compared to patients with no cognitive impairment. Decreased fractional anisotropy (FA) and increased mean diffusivity (MD) were also observed in the superior longitudinal fascicle, the inferior fronto-occipital fascicle, the striato-parietal tract, and the anterior and posterior commissural tracts. Texture analyses showed a significant difference in the right hippocampus of patients with a mixed MCI subtype.

**Conclusion**: PD-MCI patients with posterior cortical deficits have more abundant and more extensive structural alterations independently of age, disease duration, and severity, which may explain why they have an increased risk of dementia.

## Introduction

Mild cognitive impairment (MCI) is common in Parkinson’s disease (PD) since its prevalence ranges from 18.9% to 38.2% (Litvan et al., 2011). It can occur early in the course of the disease (Yarnall et al., 2014), and patients with PD and MCI (PD-MCI) have a higher risk of developing dementia (Nicoletti et al., 2019). Impaired cognitive domains are heterogeneous. Attention/working memory, executive functions, episodic memory, visuospatial functions, or language can be affected individually or in variable combinations (Caviness et al., 2007; Lawrence et al., 2016). Based on a longitudinal cohort study (the CamPaIGN cohort; Foltynie et al., 2004), Williams-Gray et al. (2009) have identified two distinct cognitive syndromes in PD using a data-driven approach: (1) a frontostriatal one, which is characterized by deficits in attention and/or executive functions and seems to be related to dopaminergic dysfunction and modulated by catechol-*O*-methyltransferase polymorphism, (2) and a posterior cortical one, which is characterized by deficits in visuospatial functions, memory encoding, and/or language and seems to have a non-dopaminergic origin and to be influenced by the microtubule-associated protein tau haplotype. This finding led to the formulation of the “dual syndrome hypothesis” in PD (Kehagia et al., 2013). Interestingly, the frontostriatal syndrome was not predictive of Parkinson’s disease dementia (PDD), whereas the posterior cortical syndrome was (Williams-Gray et al., 2009; Compta et al., 2013). Identifying PD-MCI subtypes can thus aid in the earlier detection and proactive treatment of dementia in PD. Up to now, no study has explored structural markers associated with each PD-MCI subtype defined according to the dual syndrome hypothesis.

Neuroimaging studies have reported cortical and subcortical gray matter (GM) changes and white matter (WM) tract alterations in PD-MCI compared to PD patients with normal cognition (PD-NC) and healthy controls (see Mak et al., 2015 for a review). However, the patterns of changes are inconsistent in PD-MCI. Moreover, few studies have considered the heterogeneity of impaired cognitive functions when exploring structural brain changes in PD-MCI (Bayram et al., 2019; Chen et al., 2019; Chung et al., 2019). Recently, Bayram et al. (2019) have assessed GM integrity in distinct cognitive clusters of PD patients. They were classified as having strong, typical, or weak performance in different cognitive domains. Atrophy in the frontotemporal, parietal, occipital, insular, and subcortical regions was found in patients with weak performance without specifying whether they met the criteria of PD-MCI. Furthermore, Chung et al. (2019) reported that, compared to healthy controls (HCs), PD-MCI patients with single- or multi-domain amnestic PD-MCI had cortical thinning in the left frontotemporal regions, a tendency toward a lower hippocampal volume, and a higher risk of converting to PDD. Chen et al. (2019) have shown WM tract alterations in several cerebral regions in amnestic PD-MCI compared to PD-NC patients. However, a non-negligible proportion of patients in these studies had multiple-domain PD-MCI, associating posterior cortical and frontostriatal deficits. None of these subtyping methods was based on the dual syndrome hypothesis (Kehagia et al., 2013).

The aim of this study was to identify structural changes associated with each PD-MCI subtype defined according to the dual syndrome hypothesis (Kehagia et al., 2013). No healthy control group was included in this study because we aimed at investigating cognition and its neural correlates in PD and not PD itself as a disorder. Therefore, we used a cognitively intact PD patient group (PD-NC) as a control group. Our hypothesis was that patients with PD-MCI would display structural alterations compared with PD-NC patients, particularly patients with posterior cortical deficits who are at higher risk of developing PDD. In a second step, the PD-MCI subtypes were compared with each other.

## Materials and Methods

### Participants

We used data from 156 consecutive PD patients recruited among outpatients of two university hospital movement disorder centers in Lille, France (*n* = 82), and Maastricht, The Netherlands (*n* = 76), between March 2013 and August 2014 (Dujardin et al., 2015). The inclusion criteria were: (1) meeting the United Kingdom Brain Bank criteria for idiopathic PD (Gibb and Lees, 1988) and (2) not suffering from a neurological disease other than PD. For the present study, patients having moderate to severe dementia defined as a score >1 on the Clinical Dementia Rating (Morris, 1993) and meeting the Movement Disorders Society (MDS) criteria for PDD (Emre et al., 2007) were excluded.

All participants gave their informed consent to participation in the study, which had been approved by the local institutional review boards (Lille: CPP Nord-Ouest IV, 2012-A 01317-36; Maastricht: METC azM/UM, NL42701.068.12; ClinicalTrials.gov identifier: NCT01792843).

### Demographic and Clinical Variables

The age, sex, and duration of formal education as well as PD-related variables such as disease duration, age at onset, and side of onset were recorded. All antiparkinsonian medications were recorded and the doses were converted to levodopa equivalent daily dose (Tomlinson et al., 2010). The severity of motor symptoms was assessed by part 3 of the MDS—Unified Parkinson Disease Rating Scale (MDS-UPDRS; Goetz et al., 2008), the presence of hallucinations by part 1, item 2 of the latter scale, and disease severity by the Hoehn and Yahr stage. The severity of depression, anxiety, and apathy symptoms were assessed by the 17-item Hamilton Depression Rating Scale (Hamilton, 1960), the Parkinson Anxiety Scale (Leentjens et al., 2014), and the Lille Apathy Rating Scale (Sockeel et al., 2006), respectively.

We also checked for the presence (yes/no) of high blood pressure, diabetes, hypercholesterolemia, history of lower limb arteriopathy and stroke, and sleep apnea syndrome. The frequency of clinical signs of rapid eye movement sleep behavior disorders was also recorded, as were frequencies of treatment by antipsychotic, antidepressant, or benzodiazepine.

### Neuropsychological Assessment

All patients underwent a comprehensive neuropsychological assessment including the Mattis Dementia Rating Scale (Mattis, 1976) for global cognition and standardized tests evaluating five cognitive domains: (1) attention and working memory using the forward and backward digit spans (Wechsler, 1981) and the oral version of the symbol digit modalities test (Smith, 1982); (2) executive functions using the trail making test (Reitan, 1992), a 50-item version of the Stroop word color test, and a 60-s phonemic word generation task performed in single and alternating conditions; (3) verbal episodic memory using the Hopkins verbal learning test—revised (Brandt and Benedict, 2001); (4) visuospatial functions using the Benton judgment of line orientation test—short version (Benton et al., 1978); and (5) language using the 15-item version of the Boston naming test (Graves et al., 2004). Patients were assessed in the ON state, after having received their usual antiparkinsonian medication.

### Categorization of Subtypes of Mild Cognitive Impairment

Based on the scores of the neuropsychological tests, a cognitive domain was considered impaired when performance on at least two tests was below the fifth percentile of normative data or at 1.645 standard deviations (SD) below the population mean^1^, or when one test was failed if only one was available to assess the domain. Hence, we were able to determine whether a patient had PD-MCI or not according to the MDS level I criteria for PD-MCI (Litvan et al., 2012). It is worth noting that we characterized the episodic memory deficit according to the interpretation model proposed by Brandt and Benedict (2001). The deficit was characterized either as an encoding/storage deficit or as a retrieval deficit based on various parameters from the Hopkins verbal learning test—revised (Brandt and Benedict, 2001). Only the former was considered as a sign of verbal episodic memory impairment.

Thereafter, each patient was categorized into one of the four possible cognitive subtypes: (1) normal cognition (PD-NC), i.e., no cognitive domain impaired; (2) frontostriatal subtype (PD-FS), i.e., attention/working memory and/or executive functions were impaired without deficits in visuospatial functions, episodic memory, and language; (3) posterior cortical subtype (PD-PC), i.e., visuospatial functions and/or episodic memory and/or language was impaired without deficits in attention/working memory and executive functions; (4) mixed subtype (PD-MS), i.e., attention/working memory and/or executive functions were impaired in addition to visuospatial functions and/or episodic memory and/or language deficits.

### MRI Acquisition

Patients were scanned at two sites (Lille and Maastricht) using identical 3T Philips Achieva magnetic resonance imaging (MRI) scanners (Philips Healthcare, Best, The Netherlands) with identical software versions and MR sequences. The imaging protocol included an anatomical three-dimensional T1-weighted sequence [voxel size = 1 × 1 × 1 mm^3^, repetition time (TR) = 7.2 ms, echo time (TE) = 3.3 ms, matrix size = 256 × 256 × 176 voxels, flip angle = 9°] and a diffusion tensor imaging (DTI) sequence (voxel size = 2 × 2 × 2 mm^3^, TR = 13,000 ms, TE = 55 ms, matrix size = 128 × 128 × 66 voxels, flip angle = 90°, 64 gradient directions at *b* = 1,000 s/mm^2^). To correct B0 field inhomogeneity-induced distortion, two non-diffusion-weighted images (*b* = 0 s/mm^2^) with opposite phase-encoding directions were also collected (Holland et al., 2010). For quality control, all images were visually inspected by a board-certified neuroradiologist (GK).

### MRI Data Processing

#### Cortical Regions

##### Cortical Thickness

To study cortical thinning, three-dimensional T1-weighted images were processed using the Freesurfer software version 5.3^2^. The cortical surface pipeline is described in Dale et al. (1999) and Fischl et al. (1999). Triangulated surface models of the inner and outer cortical surfaces were obtained for each patient. To avoid any segmentation errors, cortical ribbon masks were visually checked for both brain hemispheres and no manual correction of the cortical ribbon was needed. Then, the models were inflated and registered to a spherical surface atlas using a non-rigid spherical registration procedure. Cortical thickness (CTh) was computed using the t-link method, which is the Euclidean distance between the linked vertices on the inner and outer surfaces (Fischl, 2012). CTh maps were smoothed with a Gaussian surface kernel of 20 mm, full width at half maximum. The global average thickness for both hemispheres was computed as a measure of global atrophy (Segura et al., 2014).

#### Subcortical Structures

##### Subcortical Volumes

Because they are key structures in cognitive functioning, the volumes of the caudate nucleus, the thalamus, and the hippocampus in each hemisphere were segmented using volBrain^3^, a free online brain volumetry system (Manjón and Coupé, 2016). To avoid any segmentation errors, masks of each structure were manually checked and corrected.

##### Shape Analysis

To study possible surface deformations, shape analysis was performed using the SPHARM-PDM toolbox in 3D Slicer version 4.5.0–1 (Fedorov et al., 2012) on the caudate nucleus, thalamus, and hippocampus masks obtained from the volBrain segmentation. We applied the spherical harmonic-point discrimination model (SPHARM-PDM; Styner et al., 2006). Briefly, the masks were converted to 3D spherical harmonic functions, sampled into triangulated surface meshes, and aligned using a rigid-body Procrustes alignment with a mean template created from the whole sample. 15 harmonics were computed to achieve the best compromise between mesh smoothness and precision, generating 1,002 corresponding vertices on each surface.

##### Texture Analysis

To study structural parameters less sensitive to segmentation accuracy, texture analyses were performed on the left and right caudate nuclei, thalami, and hippocampi. Detailed presentation of the methodology can be found in Betrouni et al. (2020). Briefly, from the binary masks of each structure obtained from the volBrain segmentation, we have computed four first-order features to study the gray-level distribution in all voxels composing a given structure without considering spatial relationships, namely, the mean, the standard deviation, the kurtosis, and the skewness. Then, second-order features derived from the gray-level co-occurrence matrix (Haralick et al., 1973) were computed to describe the spatial relationships between the gray-level values. In this study, co-occurrence matrices were built using 26 connected directions and a distance *D* set to 1 voxel. Seven features were thus considered: contrast (i.e., the degree to which the texture intensity levels differ between voxels), entropy (i.e., the degree of uncertainty); correlation (i.e., the degree of mutual dependency between voxels); variance (i.e., gives high weights for the elements different from the average value); sum average (i.e., measures the relationship between occurrences of pairs with lower intensity values); sum variance (i.e., represents the global variation in the sum of the gray levels of voxel pair distributions); and inverse difference moments (i.e., measure the differences between the highest and the lowest values of a contiguous set of voxels).

#### White Matter Fiber Tractometry

This analysis included three steps:

- Preprocessing: the DTI data were corrected for eddy currents and geometrical and signal distortions (Glasser et al., 2013). Eddy current artifacts were corrected using the eddy_correct function in the FMRIB Software Library^4^. Then, the distortion field, inherent to echo planar images (EPI) in the phase encoding direction and responsible for geometric and signal artifacts, was calculated using a pair of spin echo EPI scans with opposite phase encoding directions (Holland et al., 2010). The “epiunwarp” function in the Computational Morphometry Toolkit (CMTK 3.2.2^5^) was used to estimate the distortion field and applied it to the DTI data.
- WM tractography: preprocessed DTI data were analyzed using TractSeg (Wasserthal et al., 2018). This method uses a supervised learning approach with a convolutional neural network to directly segment 72 common tracts. From those segmentations, the MRtrix software (v3.0^6^) was used to generate tracts using a probabilistic tracking algorithm; other parameters were set as follows: step size = 0.1 mm, minimum radius curvature = 1 mm, FOD cutoff = 0.1, minimum length = 10 mm, maximum number seeds = 2 M (Tournier et al., 2012).
- Tractometry: to estimate the integrity of the WM tracts, fractional anisotropy (FA) and mean diffusivity (MD) maps were computed for each subject. FA represents a common measurement used in DTI studies and ranges from 0 = isotropic movement of water molecules (e.g., cerebrospinal fluid) to 1 = anisotropic movement of water molecules (e.g., fiber tracts). Inversely, MD, describing the average mobility of water molecules, will be higher in the cerebrospinal fluid (approx. 3 × 10^−3^ mm^2^/s) than in white matter (approx. 5 × 10^−4^ mm^2^/s). The metrics (FA and MD) were sampled along tracts (one segment per millimeter) and then averaged weighted by its distance, allowing the analysis of the FA and MD profiles along tracts (Yeatman et al., 2012).

### Statistical Analyses

#### Neuropsychological, Demographic, and Clinical Variables

Statistical analyses were performed with the R software version 3.5.1^7^ (R Core Team, 2018). Numerical variables were described as means and standard deviations and the categorical variables as frequencies and percentages. Group comparisons were performed using Kruskal–Wallis non-parametric test and *post hoc* comparisons using Wilcoxon–Mann–Whitney test for neuropsychological data. Regarding the demographic and clinical variables, group comparisons were performed using multivariate analysis of variance (MANOVA) for numerical variables and Scheffé tests were used for *post hoc* comparisons. Categorical variables were compared with Fisher’s exact test. Multiple comparisons correction was applied with a false discovery rate (FDR) fixed at 0.05 (Benjamini and Hochberg, 1995). Adjusted *p* values <0.05 were considered significant.

#### MRI Analyses

Statistical analyses were performed using a non-parametric approach. The effect of group factor was analyzed using multivariate analysis of covariance (MANCOVA) for each MRI parameter. If significant, pairwise *post hoc* comparisons were performed. Multiple comparisons correction was applied at two levels: (a) with a permutation test (maximum number of permutations = 10,000) and (b) with a FDR fixed at 0.05. All analyses were controlled for age, sex, education duration, and recruitment center. Adjusted *p*-values <0.05 were considered significant.

In volumetric analyses, the volumes were normalized by the total intracranial volume computed from volBrain mask. In the shape analysis, each point in a patient’s mesh was scaled with the total intracranial volume of this patient. In the texture analysis, given the large number of variables (six brain structures, 11 features by structure), we used a feature selection strategy similar to that of Betrouni et al. (2020) to reduce the dimensionality. Spearman’s correlation coefficients were computed between the texture features and the PD-MCI subtypes for each structure. Features with *p* < 0.05 were considered for further analyses. In tractometric analyses, we performed statistical analyses on the mean FA and MD values of the whole tracts and on the FA and MD profiles along a tract. For the latter, to take into account the spatial information of the profiles, only clusters of *n* ≥ 5 consecutive significant segments were considered significant. *Post hoc* comparisons were performed on the mean FA or MD values computed in a given significant cluster. Regarding correlation analyses, raw scores from each cognitive parameter were transformed into *z*-scores based on normative data, and a mean *z*-score was calculated for each cognitive function (Supplementary Table 1). Partial correlation analyses using Spearman’s method were performed using the *ppcor* package (Kim, 2015) with the R software. For the shape analysis results, the distance between a participant’s mesh and the mean mesh was computed at each surface vertex, and mean distances were computed on all points (*n* = 1,002). For the texture results, values of significant features on the whole structure were used. For the tractometry results, the mean FA and MD values were computed on the whole tract for each participant. Moreover, to better understand the relationship between the measures on subcortical structures, partial correlations were also computed between the shape, texture, and subcortical volumes normalized by the total intracranial volume.

## Results

### Population

**Figure 1:**
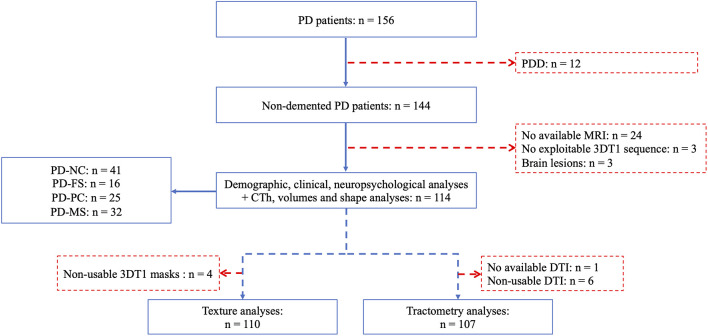
Flowchart of the study population. *PD*, Parkinson’s disease; *PDD*, Parkinson’s disease dementia; *PD-NC*, PD—normal cognition; *PD-FS*, PD—frontostriatal subtype; *PD-PC*, PD—posterior cortical subtype; *PD-MS*, PD—mixed subtype; *3DT1*, 3-dimensional T1-weighted; *CTh*, cortical thickness; *MRI*, magnetic resonance imaging; *DTI*, diffusion tensor imaging.

The flowchart of the study is shown in Figure 1. Twelve patients meeting the criteria for PD dementia were excluded as well as 30 patients who had no available or exploitable MRI scans. Consequently, analyses were performed on 114 patients [80 men; mean age= 64.77 (8.27) years, mean disease duration = 8.46 (5.86) years, mean Hoehn and Yahr stage = 2.11 (0.59), mean MDS-UPDRS motor score (part 3) = 28.89 (12.95)]. Texture and tractometric analyses were performed on subsamples similar to the main sample (see Supplementary Tables 2,3 for details).

### PD-MCI Subtypes Categorization

**Figure 2:**
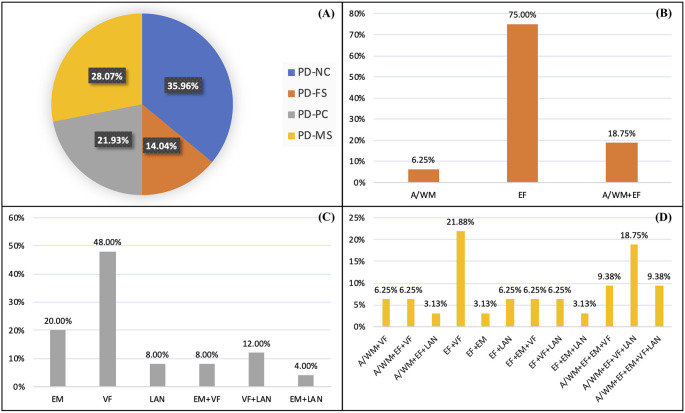
Percentages of patients in each cognitive profile **(A)** and percentages of impaired patients for each cognitive function and combination in the PD-FS **(B)**, PD-PC **(C)**, and PD-MS **(D)** subtypes. *PD-NC*, Parkinson’s disease—normal cognition; *PD-FS*, Parkinson’s disease—frontostriatal subtype; *PD-PC*, Parkinson’s disease—posterior cortical subtype; *PD-MS*, Parkinson’s disease—mixed subtype; *A/WM*, attention/working memory; *EF*, executive functions; *VF*, visuospatial functions; *EM*, episodic memory; *LAN*, language.

In total, 41 (36%) patients had no cognitive impairment (PD-NC), while 73 (64%) had a PD-MCI. Among patients with PD-MCI, 16 (14%) had a PD-FS subtype, 25 (22%) a PD-PC subtype, and 32 (28%) a mixed one (PD-MS). Detailed frequencies of the impaired cognitive functions are presented in Figure 2. PD-FS patients were mainly characterized by executive function deficits (94%), PD-PC patients by visuospatial function deficits (68%), and PD-MS by a combination of executive and visuospatial function deficits (78%). The proportions of impaired patients by test for each subtype are presented in Supplementary Table 4.

### Demographic, Clinical, and Cognitive Characterization

The results of the group comparisons on the demographic and clinical variables are shown in Table 1. No demographic and clinical differences were found between PD-NC and PD-FS. PD-MS had shorter education duration than PD-NC and PD-FS. The men/women ratio was higher in PD-NC and PD-FS than in PD-MS. Besides, more patients had an ongoing treatment with benzodiazepine in PD-MS than in PD-NC and PD-PC. Scores on the Parkinson Anxiety Scale (Leentjens et al., 2014) differed, but *post hoc* analyses were not significant after multiple comparisons adjustment. Finally, compared to Lille, Maastricht included more PD-NC patients than PD-PC and PD-MS patients. Regarding cognitive variables, detailed results of the group comparisons are shown in Table 2. PD-NC had a high level of performance in all the assessed cognitive domains. PD-FS had lower scores than PD-NC and, to a lesser extent, than PD-PC in attention/working memory and executive functions. PD-PC performed lower than PD-NC in visuospatial functions and language and had more frequent encoding/storage deficits than PD-NC and PD-FS. PD-MS had a lower overall cognitive efficiency than the three other groups and poorer performance in all cognitive domains.

**Table 1:** Sociodemographic and clinical features from the study groups.

	PD-NC (*n* = 41)	PD-FS (*n* = 16)	PD-PC (*n* = 25)	PD-MS (*n* = 32)	*P*_FDR_-value	*Post hoc* test
Demographics						
Age (years)	63.27 (8.28)	65.32 (9.48)	64.64 (8.07)	66.50 (7.78)	0.278	NA
Sex (men/women ratio)	4.13	15.00	1.50	1.13	0.022*	PD-FS ≠ PD-MS
Formal education duration (years)	13.56 (4.01)	14.81 (3.53)	11.80 (2.75)	10.25 (2.58)	<0.001*	PD-NC > PD-MS; PD-FS > PD-MS
Center, Lille/Maastricht (%)	12 (29.27)/29 (70.73)	5 (31.25)/11 (68.75)	16 (64.00)/9 (36.00)	22 (68.75)/10 (31.25)	0.011*	PD-NC ≠ PD-PC; PD-NC ≠ PD-MS
Clinical characteristics						
Disease duration (years)	9.05 (7.15)	8.44 (7.06)	8.04 (4.49)	8.06 (4.32)	0.607	NA
Age at onset (years)	54.15 (10.94)	56.94 (7.72)	56.72 (8.10)	58.47 (7.37)	0.147	NA
Side of onset (left/right/bilateral/undefined)	12/20/8/1	6/8/1/1	10/12/2/1	18/13/1/0	0.455	NA
MDS-UPDRS3 score (/132)	26.83 (12.13)	31.88 (15.50)	29.16 (11.01)	29.84 (14.14)	0.588	NA
Hoehn and Yahr stage	1.96 (0.41)	2.28 (0.77)	2.04 (0.54)	2.28 (0.67)	0.145	NA
REM sleep behavior disorder (%)	10 (24.39)	8 (50.00)	8 (32.00)	10 (31.25)	0.551	NA
MDS-UPDRS 1.2—hallucinations (%)	2 (4.88)	2 (12.50)	3 (12.00)	5 (15.63)	0.638	NA
Vascular risk factors						
High blood pressure (%)	7 (17.07)	7 (43.75)	8 (32.00)	7 (21.88)	0.344	NA
Diabetes (%)	4 (9.76)	0 (0.00)	2 (8.00)	3 (9.38)	0.786	NA
Hypercholesterolemia (%)	8 (19.51)	1 (6.25)	8 (32.00)	9 (28.13)	0.413	NA
Lower limb arteriopathy (%)	2 (4.88)	0 (0.00)	3 (12.00)	1 (3.13)	0.579	NA
Stroke history (%)	4 (9.76)	0 (0.00)	2 (8.00)	2 (6.25)	0.790	NA
Sleep apnea syndrome (%)	8 (19.51)	3 (18.75)	4 (16.00)	3 (9.38)	0.723	NA
Medication						
LEDD (mg/day)	771.56 (642.11)	743.62 (535.19)	811.56 (570.48)	844.36 (488.42)	0.692	NA
Antipsychotic (%)	0 (0.00)	0 (0.00)	0 (0.00)	1 (3.13)	0.764	NA
Antidepressant (%)	6 (14.63)	3 (18.75)	2 (8.00)	6 (18.75)	0.747	NA
Benzodiazepine (%)	2 (4.88)	0 (0.00)	1 (4.00)	9 (28.13)	0.021*	PD-NC ≠ PD-MS
Neuropsychiatric						
Hamilton Depression Rating Scale (/52)	4.88 (4.27)	5.00 (2.92)	7.04 (5.65)	6.25 (4.52)	0.243	NA
Lille Apathy Rating Scale (/36)	−26.98 (6.58)	−26.06 (5.27)	−25.88 (6.73)	−23.00 (6.65)	0.054	NA
Parkinson Anxiety Scale (/48)	4.19 (5.30)	7.94 (4.96)	8.44 (6.81)	8.41 (7.02)	0.019*	NS

Results are considered significant at *p_FDR_ < 0.05. *PD-NC*, Parkinson’s disease—normal cognition; *PD-FS*, Parkinson’s disease—frontostriatal subtype; *PD-PC*, Parkinson’s disease—posterior cortical subtype; *PD-MS*, Parkinson’s disease—mixed subtype; *MDS_UPDRS3*, Movement Disorders Society sponsored revision of the Unified Parkinson’s Disease Rating Scale—Part III (severity of motor symptoms); *MDS_UPDRS 1.2*, Movement Disorders Society sponsored revision of the Unified Parkinson’s Disease Rating Scale—Part I item 2 (presence of hallucinations); *LEDD*, levodopa equivalent daily dose; *NA*, not applicable; *NS*, not significant; *FDR*, false discovery rate.

**Table 2:** Cognitive test performance from the study groups.

	PD-NC (*n* = 41)	PD-FS (*n* = 16)	PD-PC (*n* = 25)	PD-MS (*n* = 32)	*p*_FDR_-value	*Post hoc* test
**Overall efficiency**						
Mattis Dementia Rating Scale (out of 144)	140.41 (3.25)	137.94 (5.47)	139.56 (3.39)	131.50 (8.17)	<0.0001	PD-NC > PD-MS; PD-FS > PD-MS; PD-PC > PD-MS
**Attention/working memory**						
WAIS-R forward digit span (out of 14)	8.29 (1.68)	7.38 (2.00)	8.40 (2.35)	6.47 (2.30)	<0.001*	PD-NC > PD-MS; PD-PC > PD-MS
WAIS-R backward digit span (out of 14)	6.54 (1.63)	5.31 (1.58)	6.16 (1.28)	4.47 (1.85)	<0.0001*	PD-NC > PD-FS; PD-NC > PD-MS; PD-PC > PD-MS
SDMT: correct substitutions in 90 s	50.61 (8.62)	38.75 (11.92)	44.60 (9.58)	30.28 (8.25)	<0.0001*	PD-NC > PD-FS; PD-NC > PD-PC; PD-NC > PD-MS; PD-FS > PD-MS; PD-PC > PD-MS
**Executive functions**						
Trail making test (time B)	78.78 (27.75)	134.31 (59.00)	100.39 (46.30)	194.78 (56.44)	<0.0001*	PD-NC > PD-FS; PD-NC > PD-MS; PD-FS > PD-MS; PD-PC > PD-MS
Stroop: interference time	52.33 (10.23)	81.30 (32.00)	61.84 (18.46)	92.83 (41.32)	<0.0001*	PD-NC > PD-FS; PD-NC > PD-MS; PD-PC > PD-FS; PD-PC > PD-MS
Stroop: errors	1.32 (5.92)	2.38 (3.24)	1.00 (1.41)	7.25 (9.73)	<0.0001*	PD-NC > PD-FS; PD-NC > PD-MS; PD-FS > PD-MS; PD-PC > PD-MS
Phonemic fluency: naming in 60 s	15.56 (5.02)	11.31 (3.79)	14.04 (3.25)	10.09 (3.67)	<0.0001*	PD-NC > PD-FS; PD-NC > PD-MS; PD-PC > PD-FS; PD-PC > PD-MS
Alternating fluency: naming in 60 s	15.24 (3.59)	8.56 (3.29)	12.20 (2.75)	7.84 (3.61)	<0.0001*	PD-NC > PD-FS; PD-NC > PD-PC; PD-NC > PD-MS; PD-PC > PD-FS; PD-PC > PD-MS
**Episodic memory**						
HVLT—Frequency of encoding/storage deficit (%)	0.00 (0.00)	0.00 (0.00)	8.00 (32)	10.00 (31.25)	<0.0001*	PD-NC > PD-PC; PD-NC > PD-MS; PD-FS > PD-PC; PD-FS > PD-MS
HVLT—Frequency of retrieval deficit (%)	1.00 (2.44)	1.00 (6.25)	0.00 (0.00)	4.00 (12.50)	0.131	NA
						
**Visuospatial functions**						
Judgment of line orientation (out of 15)	13.41 (1.28)	13.69 (1.20)	10.44 (2.65)	8.97 (2.51)	<0.0001*	PD-NC > PD-PC; PD-NC > PD-MS; PD-FS > PD-PC; PD-FS > PD-MS; PD-PC > PD-MS
**Language**						
Boston naming test (out of 15)	13.85 (1.15)	13.63 (1.36)	12.24 (2.52)	11.28 (2.04)	<0.0001*	PD-NC > PD-PC; PD-NC > PD-MS; PD-FS > PD-MS

Mean (and standard deviations) are presented for each cognitive parameter [except frequency (and percentages) of deficit for the HVLT]. Results are considered significant at *p_FDR_ < 0.05. *PD-NC*, Parkinson’s disease—normal cognition; *PD-FS*, Parkinson’s disease—frontostriatal subtype; *PD-PC*, Parkinson’s disease—posterior cortical subtype; *PD-MS*, Parkinson’s disease—mixed subtype; *WAIS-R*, Wechsler for adults intelligence scale—revised; *SDMT*, symbol digit modalities test; *HVLT*, Hopkins verbal learning test—revised; *NA*, not applicable; *FDR*, false discovery rate.

### MRI Analyses

There was no significant between-group difference for cortical thickness. Global cortical thickness also did not differ (Supplementary Table 5).

For the subcortical volumes, no significant between-group differences on the caudate nuclei, thalami, and hippocampi were found. Detailed volumes and statistical analysis are shown in Supplementary Table 5.

**Figure 3:**
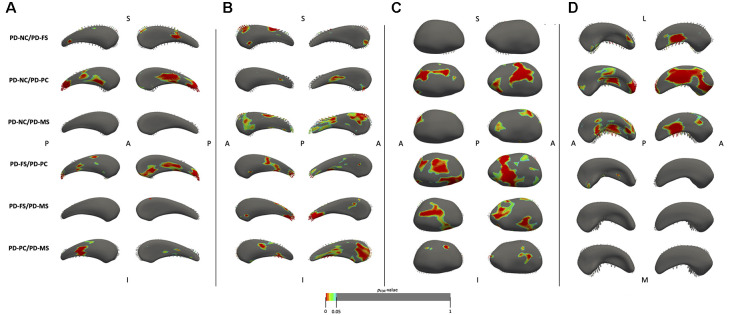
Results of the shape analysis for the left **(A)** and right **(B)** caudate nuclei, the right thalamus **(C)**, and the right hippocampus **(D)**. For the caudate nuclei and thalamus, medial face is shown on the *left* and lateral face on the *right*. For the hippocampus, superior face is shown on the *left* and inferior face on the *right*. Vectors represent the magnitude and direction of deformation at each point (only vectors pointing outward are visible). Vectors are pointing from the second group mean shape to the first group mean shape (e.g., in PD-NC/PD-FS comparison, they are pointing from PD-FS to PD-NC). Results are displayed on the mean mesh built from all patients and are considered significant at *p*_FDR_ < 0.05, corrected for age, sex, years of formal education, and center. One contrast is displayed per row. *PD-NC*, Parkinson’s disease—normal cognition; *PD-FS*, Parkinson’s disease—frontostriatal subtype; *PD-PC*, Parkinson’s disease—posterior cortical subtype; *PD-MS*, Parkinson’s disease—mixed subtype; *S*, superior; *I*, inferior; *A*, anterior; *P*, posterior; *L*, lateral; *M*, medial; *FDR*, false discovery rate.

For the shape analysis, a significant group effect was found for the bilateral caudate nucleus, right thalamus, and right hippocampus. Results of the between-group comparisons are shown in Figure 3. Compared to PD-NC, PD-FS had small local deformation fields in the left caudate nucleus (anterior and lateral regions), the right caudate nucleus (anterior and superior regions), and the right hippocampus (medio-inferior regions). Compared to PD-NC, PD-PC had large deformation fields in the left caudate nucleus (medial, lateral, and posterior regions), the right thalamus (lateral, medial, and postero-inferior regions), and the right hippocampus (antero-inferior, inferior, medio-superior, and posterior regions), while only small deformation fields were found in the right caudate nucleus (lateral regions). Compared to PD-NC, PD-MS had large deformation fields in the right caudate nucleus (antero-superior and postero-superior regions) and the right hippocampus (medio-superior, medio-inferior, and latero-posterior regions), while there were only small deformation fields in the right thalamus (antero-superior and latero-posterior regions) and no field in the left caudate nucleus. The PD-FS/PD-PC comparison showed large deformation fields in the left caudate nucleus (lateral, medial, and posterior regions) and in the right thalamus (medial, lateral, and inferior regions) and small fields in the right caudate nucleus (medial, lateral, and posterior regions) and in the right hippocampus (antero- and postero-medial regions). Regarding the PD-FS/PD-MS comparison, there were large deformation fields in the right thalamus (medial and lateral regions) and in the right caudate nucleus (postero-superior regions), while there were no significant differences in the left caudate nucleus and the right hippocampus. Finally, PD-PC/PD-MS revealed large deformation fields in the right caudate nucleus (anterior, lateral, and medial regions) and small fields in the left caudate nucleus (postero-medial and lateral regions) and in the right thalamus (antero-lateral and medio-superior regions), while there was no significant difference in the right hippocampus.

**Figure 4:**
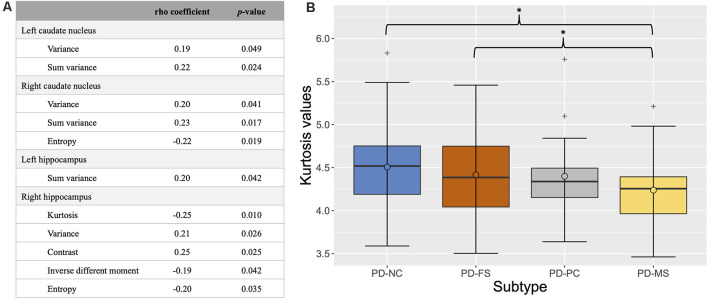
Results of the feature selection strategy **(A)** and box plot of the kurtosis values in the right hippocampus according to cognitive subtype **(B)**. In **(A)**, only significant features are shown (*p* < 0.05). In **(B)**, the *thick bars* represent the median, the *circles* the mean, and the *crosses* the outliers. *p_FDR_ < 0.05 (corrected for age, sex, years of formal education, and center). *PD-NC*, Parkinson’s disease—normal cognition; *PD-FS*, Parkinson’s disease—frontostriatal subtype; *PD-PC*, Parkinson’s disease—posterior cortical subtype; *PD-MS*, Parkinson’s disease—mixed subtype; *FDR*, false discovery rate.

For texture, the feature selection strategy showed significant correlations between groups and several features in the left and right caudate nuclei and hippocampi (see Figure 4A for details). Between-group comparisons showed a significant difference for kurtosis (*p*_FDR_ < 0.0001) in the right hippocampus. *Post hoc* analyses revealed lower kurtosis values in the right hippocampus of PD-MS compared to PD-NC and PD-FS (Figure 4B).

**Figure 5:**
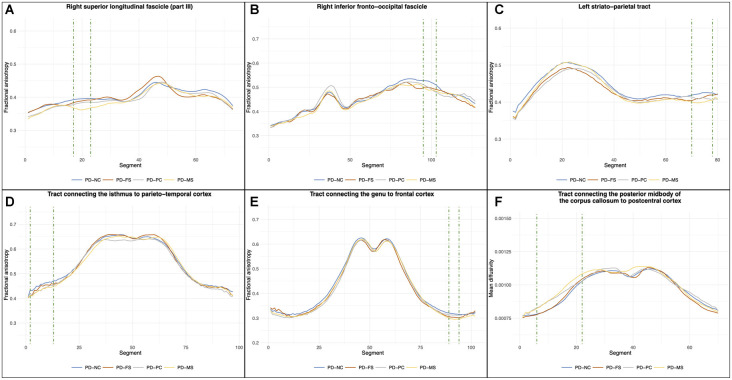
Results of the between-group comparisons on tractometric features. Position 0 corresponds to the anterior part of the fascicle for **(A)** and **(B)** and to the subcortical level for **(C)**. For **(D–F)**, position 0 corresponds to the left cortical level and the last segment corresponds to the right cortical level (with the middle corresponding to the corpus callosum part). *Green dashed line* highlights the cluster of significant segments (*p*_FDR_ < 0.05, corrected for age, sex, years of formal education, and center). *PD-NC*, Parkinson’s disease—normal cognition; *PD-FS*, Parkinson’s disease—frontostriatal subtype; *PD-PC*, Parkinson’s disease—posterior cortical subtype; *PD-MS*, Parkinson’s disease—mixed subtype; *FDR*, false discovery rate.

For WM fiber tractometry, no significant between-group differences were found on the mean FA and MD values of whole tracts. Regarding the profile analysis along tracts, the results are shown in Figure 5. These analyses revealed significant clusters of FA differences in the anterior part of the right superior longitudinal fascicle (part III), the posterior part of the right inferior fronto-occipital fascicle, the cortical level of the left striato-parietal tract, the right part of the commissural tract connecting the genu part of the corpus callosum to the frontal cortex, and the left part of the commissural tract connecting the isthmus to the parieto-temporal cortex. A significant cluster of MD difference was also found in the left part of the commissural connecting the posterior midbody of the corpus callosum to the postcentral cortex. Details of the *post hoc* comparisons are presented in Table 3. For the PD-NC/PD-FS comparisons, *post hoc* analysis showed no significant difference. Compared to PD-NC, PD-PC had decreased FA values in the isthmus tract and the right inferior fronto-occipital fascicle. There was also a trend toward a decreased FA in the left striato-parietal tract and an increased MD in the posterior midbody of the corpus callosum tract of PD-PC compared to PD-NC. Compared to PD-NC, PD-MS had reduced FA values in all the significant FA clusters [except a tendency for the superior longitudinal fascicle (part III)] and increased MD values in the posterior midbody of the corpus callosum tract. There were no significant differences in the comparisons between the three subtypes (only trends in PD-MS compared with PD-FS and/or PD-PC).

**Table 3:** Results of the *post hoc* comparisons in the significant tractometric cluster.

	Cluster size (*k*)	PD-NC (*n* = 39)	PD-FS (*n* = 15)	PD-PC (*n* = 23)	PD-MS (*n* = 30)	*Post hoc* comparisons					
						PD-NC/PD-FS	PD-NC/PD-PC	PC-NC/PD-MS	PC-FS/PD-PC	PD-FS/PD-MS	PD-PC/PD-MS
FA—Right superior longitudinal fascicle (part III)	*k* = 7	0.395 (0.035)	0.389 (0.039)	0.348 (0.031)	0.345 (0.032)	*p*_FDR_ = 0.655	*p*_FDR_ = 0.116	*p*_FDR_ = 0.072	*p*_FDR_ = 0.255	*p*_FDR_ = 0.060	*p*_FDR_ = 0.071
FA—Right inferior fronto-occipital fascicle	*k* = 9	0.521 (0.061)	0.497 (0.031)	0.495 (0.036)	0.490 (0.054)	*p*_FDR_ = 0.390	*p*_FDR_ = 0.016*	*p*_FDR_ = 0.016*	*p*_FDR_ = 0.906	*p*_FDR_ = 0.667	*p*_FDR_ = 0.451
FA—Left striato-parietal tract	*k* = 9	0.423 (0.030)	0.412 (0.046)	0.410 (0.038)	0.401 (0.041)	*p*_FDR_ = 0.320	*p*_FDR_ = 0.065	*p*_FDR_ = 0.002*	*p*_FDR_ = 0.746	*p*_FDR_ = 0.079	*p*_FDR_ = 0.274
FA—Tract connecting the isthmus to parieto-temporal cortex	*k* = 12	0.454 (0.049)	0.443 (0.027)	0.436 (0.031)	0.433 (0.030)	*p*_FDR_ = 0.555	*p*_FDR_ = 0.026*	*p*_FDR_ = 0.006*	*p*_FDR_ = 0.348	*p*_FDR_ = 0.332	*p*_FDR_ = 0.706
FA—Tract connecting the genu to frontal cortex	*k* = 6	0.317 (0.028)	0.303 (0.018)	0.312 (0.031)	0.296 (0.022)	*p*_FDR_ = 0.171	*p*_FDR_ = 0.883	*p*_FDR_ = 0.002*	*p*_FDR_ = 0.574	*p*_FDR_ = 0.113	*p*_FDR_ = 0.090
MD—Tract connecting the posterior midbody of the corpus callosum to postcentral cortex	*k* = 17	0.881 × 10^3^ (0.875 × 10^4^)	0.891 × 10^3^ (0.574 × 10^4^)	0.927 × 10^3^ (1.231 × 10^4^)	0.950 × 10^3^ (1.083 × 10^4^)	*p*_FDR_ = 1.000	*p*_FDR_ = 0.056	*p*_FDR_ = 0.004*	*p*_FDR_ = 0.454	*p*_FDR_ = 0.062	*p*_FDR_ = 1.000

Cluster size (*k*) represents the number of consecutive segments in the cluster. Columns 3–6 present the mean (and standard deviations) FA or MD values in each cluster for each subtype. *FA*, fractional anisotropy; *MD*, mean diffusivity; *PD-NC*, Parkinson’s disease—normal cognition; *PD-FS*, Parkinson’s disease—frontostriatal subtype; *PD-PC*, Parkinson’s disease—posterior cortical subtype; *PD-MS*, Parkinson’s disease—mixed subtype; *FDR*, false discovery rate *p_FDR_ < 0.05 (corrected for age, sex, years of formal education, and center).

### Correlation Analyses

The results for the partial correlations between cognitive performance and significant MRI results are shown in Table 4. Regarding shape analysis, the mean distance between patients’ meshes and mean mesh in the right hippocampus was significantly and positively associated with executive, attentional, and memory performance. There was also a significant positive correlation between the kurtosis values in the right hippocampus and executive and visuospatial functions. Finally, the mean MD values in the tract connecting the posterior midbody of the corpus callosum to the postcentral cortex were significantly and negatively associated with visuospatial functions.

**Table 4:** Correlation analyses between the MRI results and cognitive *z*-scores.

	**EF**	**A/WM**	**EM**	**VF**	**LAN**
**Shape analysis**				
Left caudate nucleus	*r_S_* = 0.14; *p*_FDR_ = 0.690	*r_S_* = 0.009; *p*_FDR_ = 1.000	*r_S_* = 0.001; *p*_FDR_ = 0.990	*r_S_* = −0.03; *p*_FDR_ = 1.000	*r_S_* = 0.1; *p*_FDR_ = 0.760
Right caudate nucleus	*r_S_* = 0.19; *p*_FDR_ = 0.245	*r_S_* = 0.04; *p*_FDR_ = 1.000	*r_S_* = 0.01; *p*_FDR_ = 0.919	*r_S_* = −0.01; *p*_FDR_ = 1.000	*r_S_* = 0.08; *p*_FDR_ = 1.000
Right thalamus	*r_S_* = 0.13; *p*_FDR_ = 0.302	*r_S_* = 0.19; *p*_FDR_ = 0.260	*r_S_* = 0.01; *p*_FDR_ = 0.885	*r_S_* = 0.06; *p*_FDR_ = 0.630	*r_S_* = 0.16; *p*_FDR_ = 0.260
Right hippocampus	*r_S_* = 0.27; *p*_FDR_ = 0.025*	*r_S_* = 0.26; *p*_FDR_ = 0.015*	*r_S_* = 0.24; *p*_FDR_ = 0.019*	*r_S_* = 0.16; *p*_FDR_ = 0.106	*r_S_* = 0.18; *p*_FDR_ = 0.072
**Texture**				
Kurtosis—Right hippocampus	*r_S_* = 0.24; *p*_FDR_ = 0.034*	*r_S_* = 0.13; *p*_FDR_ = 0.196	*r_S_* = 0.08; *p*_FDR_ = 0.545	*r_S_* = 0.34; *p*_FDR_ = 0.002*	*r_S_* = 0.02; *p*_FDR_ = 0.824
**Tractometry**				
FA—Right superior longitudinal fascicle (part III)	*r_S_* = 0.12; *p*_FDR_ = 0.375	*r_S_* = 0.19; *p*_FDR_ = 0.255	*r_S_* = 0.04; *p*_FDR_ = 0.878	*r_S_* = 0.13; *p*_FDR_ = 0.453	*r_S_* = −0.03; *p*_FDR_ = 0.764
FA—Right inferior fronto-occipital fascicle	*r_S_* = 0.17; *p*_FDR_ = 0.530	*r_S_* = 0.16; *p*_FDR_ = 0.30	*r_S_* = −0.01; *p*_FDR_ = 0.914	*r_S_* = 0.07; *p*_FDR_ = 0.592	*r_S_* = 0.10; *p*_FDR_ = 0.584
FA—Left striato-parietal tract	*r_S_* = 0.12; *p*_FDR_ = 0.379	*r_S_* = 0.15; *p*_FDR_ = 0.360	*r_S_* = 0.09; *p*_FDR_ = 0.492	*r_S_* = 0.19; *p*_FDR_ = 0.260	*r_S_* = −0.02; *p*_FDR_ = 0.824
FA—Tract connecting the isthmus to parieto-temporal cortex	*r_S_* = 0.08; *p*_FDR_ = 1.000	*r_S_* = 0.08; *p*_FDR_ = 0.719	*r_S_* = 0.004; *p*_FDR_ = 0.971	*r_S_* = 0.08; *p*_FDR_ = 0.544	*r_S_* = 0.15; *p*_FDR_ = 0.600
FA—Tract connecting the genu to frontal cortex	*r_S_* = 0.09; *p*_FDR_ = 0.975	*r_S_* = 0.19; *p*_FDR_ = 0.305	*r_S_* = 0.002; *p*_FDR_ = 0.981	*r_S_* = 0.03; *p*_FDR_ = 0.914	*r_S_* = 0.04; *p*_FDR_ = 1.000
MD—Tract connecting the posterior midbody of the corpus callosum to postcentral cortex	*r_S_* = −0.11; *p*_FDR_ = 0.658	*r_S_* = −0.06; *p*_FDR_ = 0.650	*r_S_* = 0.02; *p*_FDR_ = 0.838	*r_S_* = −0.26; *p*_FDR_ = 0.045*	*r_S_* = −0.11; *p*_FDR_ = 0.484

Results are considered significant at *p_FDR_ < 0.05 (corrected for age, sex, years of formal education, and center). *FA*, fractional anisotropy; *MD*, mean diffusivity; *A/WM*, attention/working memory; *EF*, executive functions; *VF*, visuospatial functions; *EM*, episodic memory; *LAN*, language; *FDR*, false discovery rate.

Regarding partial correlations between the MRI measures, we found significant strong positive associations between the shape results and volumetry measures for the three subcortical structures (all *p* < 0.001; see Supplementary Table 6 for details). Texture results were not significantly associated with the right hippocampus volume (*p* = 0.506).

## Discussion

The aim of our study was to identify structural modifications associated with the PD-MCI subtypes as defined by the dual syndrome hypothesis (Kehagia et al., 2013). We observed more abundant and more extensive regional shape deformation fields in the caudate nuclei, right thalamus, and right hippocampus for the PD-PC and PD-MS subtypes compared to PD-NC, while the volumes of these nuclei did not significantly differ between groups. There were also significant shape differences between the three subtypes in the caudate nuclei and the right thalamus. Moreover, we observed significant changes in the texture of the right hippocampus in PD-MS compared with PD-NC and PD-FS. Tractometric analyses revealed significant changes in the associative and commissural tracts in PD-PC and PD-MS patients compared to PD-NC. The magnitude of the structural changes was negatively correlated with the level of cognitive performance. Moreover, these changes were independent of the demographic variables such as age and education duration as well as the clinical variables such as the disease severity or duration.

### Posterior Cortical Deficits Are Associated With Structural Changes

We found no significant between-group difference in cortical thickness and subcortical volume, but significant regional shape contractions in the PD-MCI groups compared to PD-NC. These alterations were more abundant and more extensive in PD-PC and PD-MS than in PD-FS. Modification of the texture in the right hippocampus was also found in PD-MS compared to PD-NC and PD-FS. Regarding tractometric analyses, we found significant differences on several WM tracts in the PD-PC and PD-MS subtypes compared with PD-NC and no significant difference in PD-FS. This is thus consistent with our hypothesis that PD-MCI patients would have structural alterations compared to PD-NC, particularly patients with posterior cortical deficits. Cortical thinning and reduced subcortical volume have been inconsistently reported in PD-MCI compared with PD-NC, and GM alterations were more consistently found in patients with more severe cognitive impairment (see Mak et al., 2015 for a review). For example, with the same study population as ours without the exclusion of patients with mild PDD (*n* = 12), Wolters et al. (2020) used a cluster-based categorization to determine five cognitive subtypes from PD-NC to severely impaired patients. They reported a decrease in GM volume, CTh, and folding in temporal regions, cingulate cortex, and the precuneus only in the most severely impaired group (including 50% of mild PDD) compared to PD-NC. Recently, Filippi et al. (2020) showed that, at baseline, PD-MCI and PD-NC patients who converted to PD-MCI within 4 years had only a few areas of cortical thinning compared with stable PD-NC, while PD-MCI patients who converted to PDD within 4 years had more extensive areas of cortical thinning. Interestingly, in PD-NC patients who converted to PD-MCI within 4 years, the alterations concerned posterior cortical areas. Regarding subcortical volumes, Filippi et al. (2020) reported no significant difference at baseline. Altogether, our results suggest that, compared to PD-NC, GM alterations may thus be subtle at the PD-MCI stage and not detected by classical methods such as CTh and volumetry. We show that other approaches are more sensitive. Indeed, shape analysis showed local deformation fields in the caudate nuclei, right thalamus, and right hippocampus of PD-MCI patients compared to PD-NC. Mostly all significant deformation fields referred to shape contractions, suggesting regional atrophy of the subcortical nuclei in PD-MCI. Interestingly, regardless of the structure, the alterations were more abundant and more extensive in the PD-PC and PD-MS subtypes, both having posterior cortical deficits, while PD-FS patients had only small regional contraction fields compared with PD-NC. Regarding comparisons between the PD-MCI subtypes, large deformation fields were found in the caudate nuclei and the right thalamus in PD-PC and PD-MS compared with PD-FS, while there were fewer significant differences between PD-PC and PD-MS patients. These results suggest that shape analysis of the caudate nuclei and thalami could distinguish patients with frontostriatal and posterior cortical deficits (isolated or not). The shape results were all significantly and positively associated with structure volumes. Shape analysis seems thus sensitive enough for identifying regional atrophy. In a longitudinal study, Chung et al. (2017) reported that patients with PD-MCI who converted to PDD had regional contraction fields in bilateral thalamus, the right caudate nucleus, and the right hippocampus compared to PD-MCI non-converters. Moreover, Filippi et al. (2020) showed that PD-MCI patients who converted to PDD within 4 years had significant volume loss of the right thalamus and hippocampus over time. Our results show that PD-MCI subtypes with posterior cortical deficits have MRI markers of regional atrophy of GM nuclei. This suggests that neuronal damage of these nuclei is more abundant and more extensive in this subtype. This could explain why patients with this subtypes have a higher risk of converting to PDD.

Moreover, texture analysis showed significant differences in PD-MS compared with PD-NC and PD-FS on the kurtosis feature in the right hippocampus. There was no significant difference between PD-NC or PD-FS and PD-PC patients, even if the mean kurtosis value in PD-PC was lower. To our knowledge, only one study of our group has applied this method to PD-MCI. Using the same study population as ours without the exclusion of patients with mild PDD (*n* = 12) and a cluster-based categorization, Betrouni et al. (2020) have shown that the skewness and entropy parameters in the hippocampi, left thalamus, and right amygdala were able to discriminate clusters of PD patients who differed in terms of severity of the cognitive deficits, while there was no significant between-group difference in the volumes of these structures. Our results are in line with this previous study given that patients with a PD-MS subtype had more severe impairment than the others and exhibited texture changes in the right hippocampus. Sørensen et al. (2016) reported that texture changes in the hippocampi of patients with MCI without PD were more predictive of conversion to Alzheimer’s disease within 24 months than are volume changes. Hence, our result suggests that texture analysis may be a useful method to detecting structural changes earlier than the classic volumetry method, but that this discriminative potential occurs only when there is a gradient of severity between subtypes. Furthermore, the kurtosis feature, which quantifies the asymmetry of the MRI signal distribution in a given structure, has been associated with the protein tau burden in a rodent model of Alzheimer’s disease (Colgan et al., 2017). Despite further studies being needed for deciphering the link between texture and histological features in PD, it seems that this feature could be an easily available marker of neuronal damage.

Regarding tractometric analyses, our results revealed decreased FA values in the PD-PC and PD-MS subtypes compared to PD-NC along with increased MD values in PD-MS and a tendency in PD-PC. Decreased FA and increased MD values are markers of WM alterations, even if the underlying mechanisms are still debated (Alexander et al., 2007; Alba-Ferrara and de Erausquin, 2013). There were no significant differences between the three MCI subtypes, but a continuum PD-NC/PD-FS/PD-PC/PD-MS was observed for all tracts (except the genu). Few studies have considered the heterogeneity of cognitive impairment when analyzing WM alterations in PD-MCI. Recently, Chen et al. (2019) reported decreased FA in the right thalamic radiation, tapetum, and corona radiata of patients with amnestic PD-MCI compared with PD-NC, but these differences were not significant after correction for motor severity. Our results suggest that patients with posterior cortical deficits, isolated or not, have WM tract alterations that certainly contribute to the higher risk of PDD in these patients. These alterations were only found when using an accurate approach consisting of analyzing the FA/MD profiles along a tract. Interestingly, we found significant WM tract alterations at the proximity of the cortex, while cortical thickness was not affected. Hattori et al. (2012) reported WM changes in PD-MCI and PDD patients, but only the latter had GM atrophy. Rektor et al. (2018) found significant WM alterations without GM changes in PD patients compared to HCs. This suggests that WM alterations may precede GM changes and may be early markers of cognitive impairment. It is not surprising that our results were mostly located in tracts connecting posterior brain areas in both the left and right hemispheres given that these areas play an important role in the cognitive functions impaired in the PD-PC and PD-MS subtypes. Nevertheless, decreased FA values in PD-MS compared to PD-NC were also found in the right part of the tract passing through the genu of the corpus callosum, and a trend was observed for the right superior longitudinal fascicle—part III. This significant difference in the most cognitively impaired patients is in line with the study of Bledsoe et al. (2018), which reported WM tract alterations in the anterior segments of the corpus callosum of PDD patients compared to PD-NC, but no significant difference between PD-MCI and PD-NC patients. These results suggest that WM tract alterations in anterior brain areas are associated with more severe cognitive impairment. Taken together, our results showed that, in PD-MCI, not only the nature of cognitive deficits has to be considered but also their severity.

### Structural Changes Are Not Related to a Specific Anatomical Pattern

Our results showed that attentional/executive deficits are not only associated with changes in the frontal regions and the striatum and visuospatial/memory/language deficits with changes in the posterior cortical regions and the hippocampi, as could be expected from the labels used by the dual syndrome hypothesis. Indeed, regional contraction fields were found in the right hippocampus of PD-FS patients and in the bilateral caudate nucleus of PD-PC patients. Moreover, correlation analyses showed that the mean shape distances, kurtosis values in the right hippocampus, and the mean MD values were positively (negatively for MD) associated with performance in several cognitive domains. The PD-MCI subtypes were determined based on performance in neuropsychological tests that assess cognitive functions and not directly anatomical regions. The limits of the localist approach in neuropsychology (one function associated with one region) have been largely demonstrated, especially when lesions involve brain regions that are part of functional networks (Sutterer and Tranel, 2017; Warren et al., 2017). It suggests that cognitive functions involved a multitude of brain regions and that the network-based brain organization has to be considered when cognitive impairment and anatomical location are analyzed together. For example, Lang et al. (2019) reported that posterior cortical deficits were significantly associated with reduced connectivity in the central executive network. Executive functions are involved in many cognitive processes, and they likely rely on a wide range of brain regions. Therefore, the PD-MCI subtypes described in the dual syndrome hypothesis (Kehagia et al., 2013) are relevant, but using anatomical labels to refer to these subtypes seems inappropriate. We recommend using more pragmatic labels referring to the nature of cognitive deficits to avoid confusion: attention and/or executive deficits on the one hand, visuospatial, memory encoding/storage, and/or denomination deficits on the other hand. This latter subtype had more abundant and more extensive structural alterations, which probably contribute to an increased risk of developing PDD.

### Strengths and Limitations of the Present Study

The main strength of our study was investigating the potential of several structural brain markers to characterize PD-MCI subtypes in the context of the dual syndrome hypothesis. We used accurate and innovative methods such as shape, texture, and specific tractometric measures and identified specific changes in the different PD-MCI subtypes. Moreover, confounding variables were strictly controlled in our MRI analyses. We also used consensual diagnostic criteria for PD-MCI, which will facilitate inter-study comparisons. Lastly, the method used to determine the PD-MCI subtypes could easily be implemented in clinical practice, giving the possibility to clinicians to identify individual patients at risk of cognitive decline.

The main limitation of our study was the relatively small size of the PD-FS patient group, which could prevent revealing significant differences between this group and the PD-NC group. Further studies with bigger sample size are thus needed. Second, despite our neuropsychological test battery being comprehensive, it did not include at least two tests per cognitive domain, preventing the use of level II of the MDS PD-MCI criteria (Litvan et al., 2012). Moreover, the number of tests used to assess each cognitive domain was not equivalent, and this may have influenced our categorization, as well as the fact that several executive function scores were time-dependent while it was not the case for the other domains. Further cognitive subtyping studies thus need to take these issues into account.

## Conclusions and Perspectives

Our results revealed that, in PD, patients with visuospatial, encoding/storage, and/or denomination deficits, isolated or accompanied by attentional and/or executive deficits, had structural brain alterations at the PD-MCI stage. These alterations could contribute to the increased risk of developing PDD reported in these patients. Innovative and accurate techniques of MRI analysis are, however, needed to identify these markers. Furthermore, the structural changes were not circumscribed to regions designated by the labels (i.e., frontostriatal and posterior cortical), suggesting that using such labels to refer to cognitive subtypes is not appropriate. Further studies are needed to determine whether the structural MRI markers found in the present study are present at the early or even the prodromal stage of the disease and can be used to identify patients at risk of converting from PD-MCI to PDD.

## Data Availability Statement

The raw data supporting the conclusions of this article will be made available by the authors, without undue reservation.

## Ethics Statement

The studies involving human participants were reviewed and approved by the local institutional review boards CPP Nord-Ouest IV (2012-A 01317-36) for Lille (France) and METC azM/UM (NL42701.068.12) for Maastricht (The Netherlands). ClinicalTrials.gov identifier: NCT01792843. The patients/participants provided their written informed consent to participate in this study.

## Author Contributions

QD, KD, RL, and AL contributed to the conception and design of the study. QD and KD organized the database. QD, RV, and GC performed MRI data preprocessing. QD, RV, and NB performed MRI analyses. QD performed the statistical analysis and wrote the first draft of the manuscript. RV wrote a section of the manuscript. GK inspected all MRI images for quality control. All authors contributed to the article and approved the submitted version.

## Conflict of Interest

The authors declare that the research was conducted in the absence of any commercial or financial relationships that could be construed as a potential conflict of interest.

## Footnotes

^1^This value of SD corresponds to the fifth percentile.

^2^https://surfer.nmr.mgh.harvard.edu

^3^https://www.volbrain.upv.es/

^4^http://fsl.fmrib.ox.ac.uk/fsl/fslwiki/

^5^http://www.nitrc.org/projects/cmtk/

^6^http://www.mrtrix.org/

^7^http://www.r-project.org

## Abbreviations

CTh: cortical thickness
DTI: diffusion tensor imaging
FA: fractional anisotropy
FDR: false discovery rate
GM: gray matter
MCI: mild cognitive impairment
MD: mean diffusivity
MDS: Movement Disorder Society
MRI: magnetic resonance imaging
PD: Parkinson’s disease
PDD: Parkinson’s disease dementia
PD-MS: Parkinson’s disease mixed subtype
PD-NC: Parkinson’s disease normal cognition
PD-PC: Parkinson’s disease posterior cortical subtype
PD-FS: Parkinson’s disease frontostriatal subtype
WM: white matter.
