# Supplementary material for: Posterior Cortical Cognitive Deficits Are Associated With Structural Brain Alterations in Mild Cognitive Impairment in Parkinson’s Disease
Source: Front Aging Neurosci. 2021 May 13;13:668559. doi: 10.3389/fnagi.2021.668559 (PMC8155279; doi:10.3389/fnagi.2021.668559)
Supplement: Supplementary file 1 [file Data_Sheet_1.docx]

|  | PD-NC (n=41) | PD-FS (n=16) | PD-PC (n=25) | PD-MS (n=32) |
| --- | --- | --- | --- | --- |
| EF | 0.19 (1.11) | -1.06 (0.72) | -0.20 (0.48) | -2.32 (1.82) |
| A/WM | -0.05 (0.70) | -1.04 (0.96) | -0.41 (0.70) | -1.75 (0.71) |
| EM | -0.02 (0.88) | -0.32 (0.65) | -0.89 (1.67) | -1.43 (1.86) |
| VF | 0.91 (0.61) | 1.04 (0.57) | -0.50 (1.26) | -1.21 (1.19) |
| LAN | 0.64 (0.64) | 0.51 (0.76) | -0.26 (1.40) | -0.79 (1.13) |

**Supplementary Table 1.** Cognitive z-scores (mean and standard deviations) for each cognitive function according to cognitive profile. PD-NC = Parkinson’s disease – normal cognition; PD-FS = Parkinson’s disease – frontostriatal subtype; PD-PC = Parkinson’s disease – posterior cortical subtype; PD-MS = Parkinson’s disease – mixed subtype; A/W = attention and working memory; EF = executive functions; VF = visuospatial functions; EM = episodic memory; LAN = language.

|  | PD-NC (n=40) | PD-FS  (n=16) | PD-PC (n=23) | PD-MS (n=31) | *p*_FDR_-value | *post-hoc* test |
| --- | --- | --- | --- | --- | --- | --- |
| DEMOGRAPHICS | | | | | | |
| Age (years) | 62.97 (8.16) | 65.32 (9.48) | 64.61 (8.43) | 66.88 (7.59) | 0.096 | NA |
| Sex (men/women ratio) | 4.00 | 15.00 | 1.30 | 1.21 | 0.024* | PD-SF≠PD-PC;  PD-SF≠PD-MS |
| Formal education duration (years) | 13.45 (4.00) | 14.81 (3.53) | 11.48 (2.63) | 10.19 (2.61) | <0.001* | PD-NC>PD-MS; PD-SF>PD-MS |
| Center (Lille/Maastricht; %) | 11 (27.50)/  29 (72.50) | 5 (31.25)/  11 (68.75) | 15 (65.22)/  8 (34.78) | 21 (67.74)/  10 (32.26) | 0.005* | PD-NC≠PD-PC; PD-NC≠PD-MS |
| CLINICAL CHARACTERISTICS | | | | | | |
| Disease duration (years) | 9.23 (7.15) | 8.44 (7.06) | 8.43 (4.40) | 8.06 (4.39) | 0.414 | NA |
| Age at onset (years) | 53.68 (10.64) | 56.94 (7.72) | 56.30 (8.26) | 58.84 (7.18) | 0.048* | NS |
| Side of onset (left/right/bilateral/undefined) | 12/19/8/1 | 6/8/1/1 | 8/12/2/1 | 17/13/1/0 | 0.333 | NA |
| MDS-UPDRS3 score (/132) | 26.68 (12.24) | 31.88 (15.50) | 29.22 (11.42) | 30.39 (14.03) | 0.363 | NA |
| Hoehn & Yahr stage | 1.96 (0.41) | 2.28 (0.77) | 2.04 (0.56) | 2.29 (0.68) | 0.092 | NA |

**Supplementary Table 2.** Sociodemographic and clinical features from texture analysis subsample. Results are considered significant at ******p*_FDR_<0.05. PD-NC = Parkinson’s disease – normal cognition; PD-FS = Parkinson’s disease – frontostriatal subtype; PD-PC = Parkinson’s disease – posterior cortical subtype; PD-MS = Parkinson’s disease – mixed subtype; MDS_UPDRS3 = Movement Disorders Society sponsored revision of the Unified Parkinson's Disease Rating Scale-Part III (severity of motor symptoms); NA = Not applicable; NS = Not significant; FDR = false discovery rate.

|  | PD-NC (n=39) | PD-FS  (n=15) | PD-PC (n=23) | PD-MS (n=30) | *p*_FDR_-value | *post-hoc* test |
| --- | --- | --- | --- | --- | --- | --- |
| DEMOGRAPHICS | | | | | | |
| Age (years) | 63.69 (8.12) | 64.17 (8.55) | 64.66 (8.43) | 66.36 (7.82) | 0.284 | NA |
| Sex (men/women ratio) | 3.88 | 14.00 | 1.30 | 1.14 | 0.028* | PD-SF≠PD-MS |
| Formal education duration (years) | 13.69 (4.07) | 14.93 (3.61) | 11.74 (2.77) | 10.23 (2.66) | <0.001* | PD-NC>PD-MS; PD-SF>PD-MS |
| Center (Lille/Maastricht; %) | 12 (30.77)/  27 (69.23) | 5 (33.33)/  10 (66.67) | 14 (60.87)/  9 (39.13) | 21 (70.00)/  9 (30.00) | 0.019* | PD-NC≠PD-MS |
| CLINICAL CHARACTERISTICS | | | | | | |
| Disease duration (years) | 8.67 (7.10) | 7.20 (5.21) | 8.48 (4.37) | 8.10 (4.46) | 0.776 | NA |
| Age at onset (years) | 54.95 (10.37) | 57.07 (7.98) | 56.30 (8.26) | 58.30 (7.36) | 0.261 | NA |
| Side of onset (left/right/bilateral/undefined) | 11/19/8/1 | 6/7/1/1 | 9/11/2/1 | 17/12/1/0 | 0.323 | NA |
| MDS-UPDRS3 score (/132) | 26.49 (12.33) | 29.00 (10.75) | 28.57 (11.26) | 30.03 (14.59) | 0.292 | NA |
| Hoehn & Yahr stage | 1.96 (0.42) | 2.10 (0.28) | 2.09 (0.51) | 2.30 (0.69) | 0.027* | NS |

**Supplementary Table 3.** Sociodemographic and clinical features from tractometric analysis subsample. Results are considered significant at ******p*_FDR_<0.05. PD-NC = Parkinson’s disease – normal cognition; PD-FS = Parkinson’s disease – frontostriatal subtype; PD-PC = Parkinson’s disease – posterior cortical subtype; PD-MS = Parkinson’s disease – mixed subtype; MDS_UPDRS3 = Movement Disorders Society sponsored revision of the Unified Parkinson's Disease Rating Scale-Part III (severity of motor symptoms); NA = Not applicable; NS = Not significant; FDR = false discovery rate.

|  | PD-NC (n=41) | PD-FS (n=16) | PD-PC (n=25) | PD-MS (n=32) |
| --- | --- | --- | --- | --- |
| ATTENTION/WORKING MEMORY |  |  |  |  |
| WAIS-R forward and backward digit spans | 0 (0.00) | 5 (31.25) | 2 (8.00) | 18 (56.25) |
| SDMT | 2 (4.88) | 6 (37.50) | 8 (32.00) | 28 (87.50) |
| EXECUTIVE FUNCTIONS |  |  |  |  |
| Trail Making Test | 0 (0.00) | 3 (18.75) | 3 (12.00) | 25 (78.13) |
| Stroop | 3 (7.32) | 9 (56.25) | 3 (12.00) | 21 (65.63) |
| Phonemic fluency | 9 (21.95) | 11 (68.75) | 4 (16.00) | 15 (46.88) |
| Alternating fluency | 0 (0.00) | 10 (62.50) | 1 (4.00) | 21 (65.63) |
| EPISODIC MEMORY |  |  |  |  |
| HVLT – Encoding/storage deficit | 0 (0.00) | 0 (0.00) | 8.00 (32) | 10.00 (31.25) |
| HVLT – Retrieval deficit | 1.00 (2.44) | 1.00 (6.25) | 0.00 (0.00) | 4.00 (12.50) |
| VISUOSPATIAL FUNCTIONS |  |  |  |  |
| Judgment of line orientation | 0 (0.00) | 0 (0.00) | 17 (68.00) | 27 (84.38) |
| LANGUAGE |  |  |  |  |
| Boston naming test | 0 (0.00) | 0 (0.00) | 6 (24.00) | 15 (46.88) |

**Supplementary Table 4.** Number (and percentages) of impaired patients for each cognitive test. PD-NC = Parkinson’s disease – normal cognition; PD-FS = Parkinson’s disease – frontostriatal subtype; PD-PC = Parkinson’s disease – posterior cortical subtype; PD-MS = Parkinson’s disease – mixed subtype; WAIS-R = Wechsler for adults intelligence scale revised; SDMT = Symbol digit modalities test; HVLT = Hopkins verbal learning test-revised; FDR = false discovery rate.

|  | PD-NC  (n=41) | PD-FS  (n=16) | PD-PC  (n=25) | PD-MS  (n=32) | *p*_FDR_-value |
| --- | --- | --- | --- | --- | --- |
| Left caudate nucleus | 2312.319*10^-6^  (278.462*10^-6^) | 2152.493*10^-6^  (256.656*10^-6^) | 2287.401*10^-6^  (307.937*10^-6^) | 2272.577*10^-6^  (280.103*10^-6^) | 0.905 |
| Right caudate nucleus | 2369.997*10^-6^  (272.886*10^-6^) | 2231.851*10^-6^  (244.708*10^-6^) | 2359.818*10^-6^  (324.050*10^-6^) | 2313.619*10^-6^  (282.698*10^-6^) | 0.741 |
| Left thalamus | 3618.976*10^-6^  (334.870*10^-6^) | 3577.435*10^-6^  (320.103*10^-6^) | 3676.788*10^-6^  (279.744*10^-6^) | 3634.136*10^-6^  (373.395*10^-6^) | 1.000 |
| Right thalamus | 3573.680*10^-6^  (311.980*10^-6^) | 3579.176*10^-6^  (303.416*10^-6^) | 3695.061*10^-6^  (321.632*10^-6^) | 3559.852*10^-6^  (377.798*10^-6^) | 0.702 |
| Left hippocampus | 2716.094*10^-6^  (281.155*10^-6^) | 2580.491*10^-6^  (237.396*10^-6^) | 2684.289*10^-6^  (228.696*10^-6^) | 2613.260*10^-6^  (308.375*10^-6^) | 0.766 |
| Right hippocampus | 2770.881*10^-6^  (231.690*10^-6^) | 2657.329*10^-6^  (225.690*10^-6^) | 2726.209*10^-6^  (254.422*10^-6^) | 2652.175*10^-6^  (300.883*10^-6^) | 1.000 |
| Global cortical thickness | 2.38 (0.12) | 2.36 (0.09) | 2.36 (0.10) | 2.33 (0.12) | 0.540 |

**Supplementary Table 5.** Mean (SD) volumes (in mm^3^) of subcortical structures and global cortical thickness (in mm) according to cognitive subtype. All volumes are normalized with total intracranial volume. Results are considered significant at *p*_FDR_<0.05, corrected for age, sex, years of formal education and center. PD-NC = Parkinson’s disease – normal cognition; PD-FS = Parkinson’s disease – frontostriatal subtype; PD-PC = Parkinson’s disease – posterior cortical subtype; PD-MS = Parkinson’s disease – mixed subtype; FDR = false discovery rate.

|  | Volume –  Left caudate nucleus | Volume –  Right caudate nucleus | Volume –  Right thalamus | Volume –  Right hippocampus |
| --- | --- | --- | --- | --- |
| SHAPE ANALYSIS | | | | |
| Left caudate nucleus | rho = 0.74;  *p*_FDR_ < 0.0001* | NA | NA | NA |
| Right caudate nucleus | NA | rho = 0.73;  *p*_FDR_ < 0.0001* | NA | NA |
| Right thalamus | NA | NA | rho = 0.53;  *p*_FDR_ < 0.0001* | NA |
| Right hippocampus | NA | NA | NA | rho = 0.54;  *p*_FDR_ < 0.0001* |
| TEXTURE | | | | |
| Kurtosis – Right hippocampus | NA | NA | NA | rho = -0.07;  *p*_FDR_ = 0.506 |

**Supplementary Table 6.** Correlation analyses between volumes and shape/texture analysis on subcortical structures. Volumes were normalized by the total intracranial volume. Results are considered significant (*) at *p*_FDR_<0.05, corrected for age, gender, education and center. NA = not applicable; FDR = false discovery rate.
